# Immobilization Techniques for Microarray: Challenges and Applications

**DOI:** 10.3390/s141222208

**Published:** 2014-11-25

**Authors:** Satish Balasaheb Nimse, Keumsoo Song, Mukesh Digambar Sonawane, Danishmalik Rafiq Sayyed, Taisun Kim

**Affiliations:** 1 Institute for Applied Chemistry and Department of Chemistry, Hallym University, Chuncheon 200-702, Korea; E-Mails: satish_nimse@hallym.ac.kr (S.B.N.); mukeshsonawane87@gmail.com (M.D.S.); danishrs@gmail.com (D.R.S.); 2 Biometrix Technology, Inc. 202 BioVenture Plaza, Chuncheon 200-161, Korea; E-Mail: hanlimsk@empal.com

**Keywords:** microarray, 9G technology, biosensors, DNA self-assembly, hybridization, biomarker

## Abstract

The highly programmable positioning of molecules (biomolecules, nanoparticles, nanobeads, nanocomposites materials) on surfaces has potential applications in the fields of biosensors, biomolecular electronics, and nanodevices. However, the conventional techniques including self-assembled monolayers fail to position the molecules on the nanometer scale to produce highly organized monolayers on the surface. The present article elaborates different techniques for the immobilization of the biomolecules on the surface to produce microarrays and their diagnostic applications. The advantages and the drawbacks of various methods are compared. This article also sheds light on the applications of the different technologies for the detection and discrimination of viral/bacterial genotypes and the detection of the biomarkers. A brief survey with 115 references covering the last 10 years on the biological applications of microarrays in various fields is also provided.

## 1. Introduction

Microarrays (DNA chips) are important tools for high-throughput analysis of biomolecules [1,2]. The use of microarrays for parallel screening of nucleic acid and protein profiles has recently become an industry standard for drug discovery and biomarker identification. The success of DNA chips depends on the chemistry used for the immobilization of the DNA probes. Moreover, the success of the DNA chips also depends on the good accessibility and functionality of the surface-bound probes, the density of attachment, and the reproducibility of the attachment chemistry [3–5]. There are two types predominant methods for the construction of oligonucleotide microarrays: the direct (*in situ*) syntheses of oligonucleotides on the chip surface by using photolithographic methods and the deposition methods [6]. The *in situ* synthesis protocol allows the preparation of high-density oligonucleotide microarrays; however, it suffers from certain drawbacks [7,8]. The drawbacks of deposition methods are the decrease in the hybridization efficiency with an increase in the density of the immobilized probes and the reproducibility of the immobilization method. Furthermore, the spots on such surfaces often reveal inhomogeneous signal distribution [9–12]. The other drawbacks are the elevated hybridization temperature, the low hybridization efficiency, and longer hybridization time [13–15]. Hence, methods for the hybridization of the oligonucleotides at room temperature with high accessibility and high efficiency are important in the field of DNA chip technology [16–18].

The performance of DNA chips was under shadow due to the several issues including the probe design, the reaction conditions during spotting, the hybridization and washing conditions. Furthermore, the suppression of nonspecific binding, the distance between the oligonucleotides and the surface also add to the factors responsible for DNA chip problems. The lateral spacing between the immobilized oligonucleotides also determines the performance of the DNA chips [19]. Many research groups have noticed the unique aspect of the lateral spacing between the oligonucleotides [20–22]. The lateral spacing phenomenon is not only important to make DNA chips but also to make arrays of the proteins [23–25], aptamers [26], and small molecules using the DNA-Directed Immobilization (DDI) method [27,28]. Until now, mixed self-assembled monolayers have been generally tried to control the lateral spacing between the oligonucleotides on the Au substrates [29,30]. The immobilization of the oligonucleotides with lateral spacing not only ensures the accessibility of a target probes but also increases the hybridization yield [31]. This article elaborates a comparison of different DNA immobilization technologies used in the production of diagnostic DNA chips.

## 2. DNA Immobilization Methods

DNA probes are short oligonucleotides which can hybridize with specific target sequences. The immobilization step for the DNA probes is essential to develop a whole range of microarrays. Immobilization can be defined as the attachment of molecules to a surface resulting in reduction or loss of mobility. The way in which DNA's are immobilized determines the property of a microarray. Generally, the choice of a suitable immobilization strategy is determined by the physicochemical properties of both surface and DNA probes. DNA microarrays developed by different strategies have a common critical step of immobilization of the DNA probes on the surface. To develop microarrays, the probes can be made base-by base on the support or pre-synthesized and then spotted on the surface.

**Figure 1.**
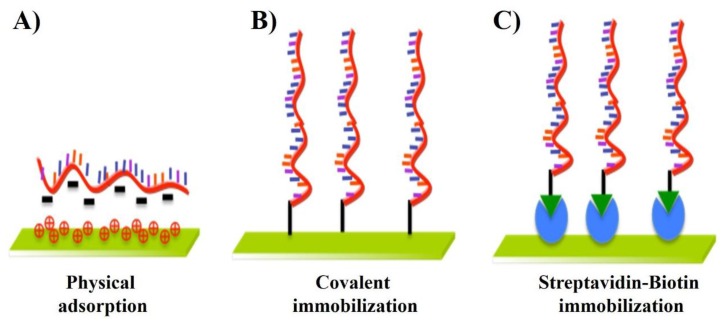
Immobilization techniques for fabrication DNA microarray.

As shown in the Figure 1 and Table 1, many immobilization techniques have been developed in the past years, which are mainly based on three important mechanisms: (A) physical adsorption; (B) covalent immobilization; and (C) streptavidin-biotin immobilization. The achievement of high sensitivity and selectivity requires minimization of nonspecific adsorption and the stability of the immobilized DNA probes. The control of this step is essential to ensure high reactivity, orientation, accessibility, and stability of the surface-confined probe and to avoid nonspecific binding. Table 1 depicts the unresolved challenges involved in the immobilization techniques.

### 2.1. Immobilization by Physical Adsorption

Physical adsorption is the simplest immobilization method because it does not require any nucleic acid modification. Immobilization has been reported based on ionic interactions occurring between the negatively charged groups present on the DNA probe and positive charges covering the surface as depicted in Table 2. The resulting immobilized DNAs are likely to be heterogeneous and randomly oriented on the surface because each molecule can form many contacts in different orientations to minimize repulsive interactions with the substrate and previously adsorbed DNA probes. For instance, a chitosan film was used for the immobilization of ssDNA on a glassy carbon electrode (GCE) by physical adsorption [39,40]. The coupling between the negatively charged phosphate backbone of the DNA probes and a positively charged film surface also allowed the development of DNA microarrays [41]. The potential applied during immobilization enhances the stability of the probe through the electrostatic attraction between the positively charge surface and the negatively charged sugar-phosphate backbone of DNA.

The limitation of the adsorption mechanism is random orientation and weak attachment of DNAs to the surface. Due to the weak attachment the DNA probes can be removed by some buffers or detergents when performing the assays. Moreover, the problems related to the mass transport effect and high background signals emanating from nonspecific interactions can result not only in the false calculation of kinetic rate constants, but also false detection of pathogenic infections.

### 2.2. Immobilization by Covalent Attachment

In the immobilization of DNA on solid-state surfaces using the electrostatic interaction as a driving force, environmental changes such as ionic strength, pH, and temperature can cause desorption of the adsorbed DNA probes. Therefore, a covalent coupling route for the immobilization of DNA probes to achieve good stability and high binding strength is more facile [37,42]. Chemisorption and covalent attachment are the two common covalent attachment methods for the immobilization of DNAs on the surface reported in the literature. The Table 2 gives information on the functional groups potentially available on the surface for DNA for immobilization.

**Figure 2.**
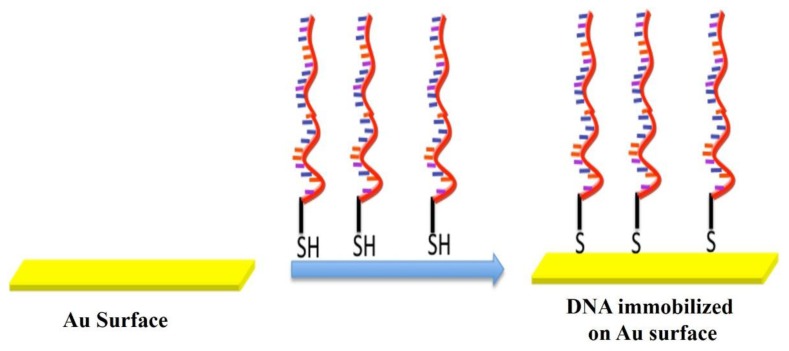
DNA immobilization on Au (Gold) surface.

As shown in Figure 2, thiol-metal interactions are frequently used for covalent binding of biomolecules on gold surfaces. The thiol groups demonstrate the strong affinity towards the noble metal surfaces allowing the formation of covalent bonds between the sulfur and gold atoms. On the basis of this principle (chemisorption), DNA microarrays have been developed using thiol-modified DNA probes. Similarly the DNA probes were immobilized on gold-interdigitated ultramicro-elecrode arrays by self-assembly of thiol-modified DNA probes [64] and also were attached to gold micropads deposited on a silicon surface [65].

The technique of immobilization of thiol-DNA probes by self-assembly on gold electrodes is widely used in the fabrication of electrochemical and DNA biosensors. The technique uses the advantage of the strong interaction (chemisorption) between thiolated DNA and gold surfaces. Self-assembled monolayers obtained by using thiol-tethered oligonucleotides mixed with alkanethiols such as mercaptohexanol represent a simple yet effective means to control the density and availability of the capture probe [66]. The secondary thiol displaces the non-specifically adsorbed probe molecules, while leaving the remaining ones in an upright position. Thus the orderly arrangement of probes results in an increased hybridization efficiency. An additional feature of this immobilisation chemistry is the stability of the surface-attached monolayer of biomolecules.

The covalent immobilization method requires chemical modification of the surface when fabricating microarrays. The SiOH groups on the glass surface are modified to either nucleophilic or electrophilic functionalities that then react with the amine or carboxylated oligonucleotide probes. Therefore, the surface chemistries for the attachment of oligonucleotides on the surfaces have been extensively investigated [67]. However, a suitable method would be the one which allows an efficient covalent attachment with a uniform probe density across the surface. Moreover, the immobilized probes should not interfere with the highly target specific hybridizations. The biomolecule attachment method should be easily transferable from the laboratory to mass production scale. Moreover, it should be reliable, repeatable, and capable of withstanding the conditions employed during the blocking and hybridization steps.

Different covalent attachment chemistries involving various functional groups on the surface are summarized in the Table 3, along with their advantages and drawbacks. Covalent reactions often use carbodiimide as a reagent. 1-Ethyl-3-(3-dimethylaminopropyl) carbodiimide (EDC) is the most frequently used activation coupling reagent, as shown in the Table 3. The water-soluble EDC-mediated reaction converts the carboxyl group into the unstable O-acylisourea intermediate that readily reacts with the amine group, resulting in covalent amide bonds between biomolecules and solid surfaces [68]. The DNA probes can be immobilized on the carboxylate terminated 4-aminobenzoic acid monolayers via EDC and N-hydroxysulfosuccinimide (Sulfo-NHS). Aminated or carboxylated DNAs can also be immobilized on the respective carboxylated or aminated single walled carbon nanotubes (SWNT) by using EDC coupling [69].

The Schiff-base reaction between aldehyde groups on the substrate surface and amine groups of DNA probes results in the covalent attachment of DNA probes on solid surfaces [70]. The interaction between amine and aldehyde groups leads to the formation of a labile Schiff's base that can be stabilized by reduction creating a stable secondary amine linkage. Moreover, the Schiff-base reaction using glutaraldehyde as a cross-linker provides a covalent bridge between the amine groups of DNA probes and amine-functionalized surfaces under moderate conditions. The ssDNA can be covalently immobilized on cantilevers using glutaraldehyde to develop DNA chips [71].

**Figure 3.**
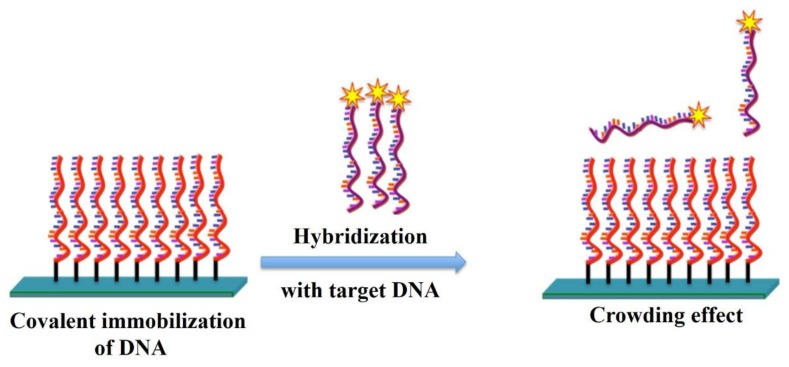
Crowding effect on the DNA-DNA hybridization due to the high immobilization density.

However, the vast majority of the immobilization chemistries described in Table 3 were designed to find optimum conditions that gives a maximum hybridization signal rather than high specificity. Factors that influence the fabrication of DNA-modified surfaces to obtain microarrays are the immobilization chemistry, spotting buffer, probe concentration. The physical factors like spotter type, pins used, and environmental conditions also play major roles in the performance of the microarray. The goal of these methods should be a microarray with evenly spaced probes over the surface at an optimal distance from each other to allow for high hybridization efficiencies and high specificity. Maximum hybridization signal and maximum hybridization efficiency are not necessarily obtained at the same probe density. The reason is that DNA probes that are too closely packed cannot participate in hybridization reaction due to steric hindrance or electrostatic interactions [72–74]. Moreover, the closely packed probes also result in a high signal to background ratio and non-specific interactions. The phenomenon of low signal intensity with high signal to background ratio and non-specific interaction due to compact arrangement of probes on the surface as shown in Figure 3 is called as crowding effect.

It was generally considered that chips with high immobilization density perform well upon hybridization with the target DNA probes. However, due to the compactness of the immobilized probes, the incoming target probes do not have enough space to get into the immobilized probes and to bind with them. Thus, many methods use a high temperature hybridization step to allow the target probe to bind with immobilized probes. The use of high temperature hybridization and the crowding effect of the immobilized probes are the main causes of low signal to background ratios, non-specific hybridization, and low sensitivity. Thus the crowding effect as depicted in the Figure 3 is the main problem faced by most immobilization methods which needs to be solved for next generation DNA chips. It is well known that a DNA immobilized surface with sufficient distance between each DNA probe could ensure high hybridization yield [75].

### 2.3. Immobilization by Streptavidin-Biotin (Avindin-Biotin) Interactions

**Figure 4.**
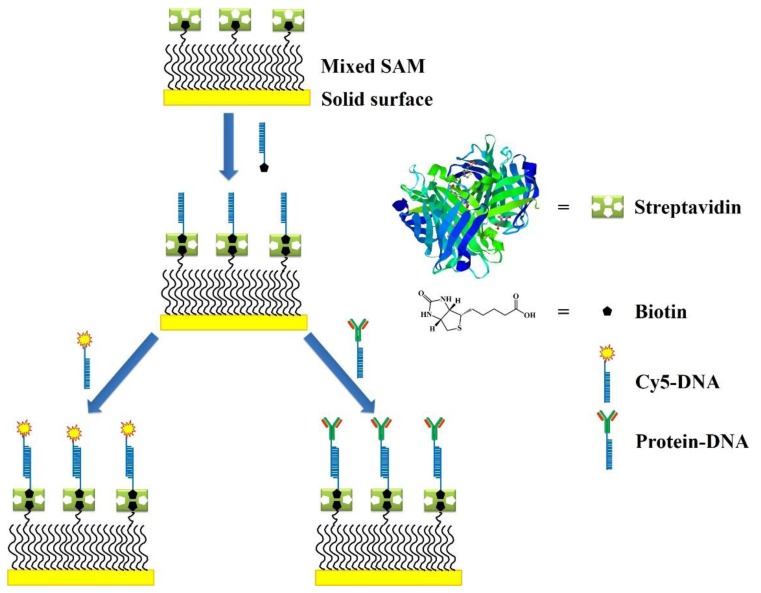
Immobilization of DNAs by using streptavidin-biotin interactions.

Various methods for the immobilization of streptavidin on the solid surfaces have been reported. The formation of the streptavidin and biotin complex is useful in a wide variety of applications [76,77]. The highly specific binding of streptavidin and biotin is largely used to immobilize DNA on the surfaces as demonstrated in the Figure 4. This is a two step method, where the the solid surface is first biotinylated using a crosslinker reagent, followed by step of streptavidin addition. Biotin is a small molecule that binds with a very high affinity to the avidin or streptavidin binding sites (*K_a_* = 10^15^ M^−1^). Moreover, avidin and streptavidin are tetrameric proteins that have four identical binding sites for biotin. Streptavidin with an isoelectric point (pI) equal to 5 is thus preferably used over avidin, which has a pI of 10.5, to avoid nonspecific interactions [78].

The avidin (or streptavidin)-biotin interaction is often used to develop DNA microarrays. DNA probes were succesfuly bound to a SAM of 2-mercaptoethanol and 11-mercaptoundecanoic acid through streptavidin-biotin interactions. Avidin can be adsorbed on the silica surface before immobilizing a biotinylated molecular beacon (MB). Three out of four sites in avidin remain free to interact with the biotinylated DNA probes [37].

Though streptavidin is easily immobilized on various surfaces the binding capacity decreases over time. Moreover, the the synthesis of streptavidin-immobilized surfaces involves multiple steps, including synthetic modification of the surface, immobilization of streptavidin, and blocking. Each step increases the production time and cost. Moreover, the immobilization of streptavidin or biotin on the suface suffers from drawbacks like the instability of the immobilized proteins and non-specific interactions, thus resulting in the low sensitivity amd specificity [79].

### 2.4. Immobilization by Using Nanocones

**Figure 5.**
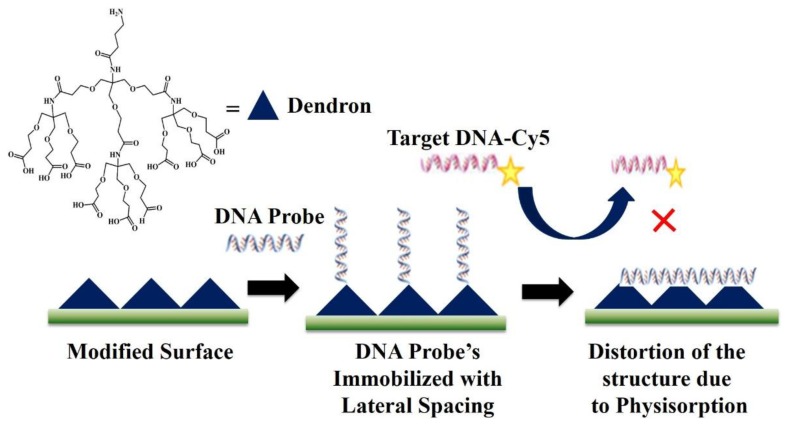
Use of nanocones (dendrons) for the covalent immobilization of DNA.

The problems due to the high compactness of immobilized prones as depicted in the Figure 3 were solved by using a cone-shaped dendron as demonstrated in Figure 5. The dendrons were successfully introduced on various oxide substrates to obtain relatively uniform *mesospacing* between the apexes of the dendron molecules. The dendron-modified substrate provided lateral spacing between the immobilized probes [80]. The problem of non-specific interactions was solved to a great extent as the incoming target DNA now has enough space to get closer to the immobilized complementary probe and bind. Moreover, the target DNA with the non-commentary sequence was washed away in the washing step as it has much less interaction with the immobilized probes. Thus, the problem of non-specific interactions was solved to a great extent by providing lateral spacing between the immobilized probes. However, as shown in the Figure 5, the drawback of this method is that immobilized probes can fall on the surface and show physisorption on the chip as they do not have a support to stand on. Due to this problem, DNA chips with Dendron-modified surfaces need a very high hybridization and washing temperatures and eventually show a very low sensitivity.

### 2.5. DNA Immobilization by Using 9G Technology

**Figure 6.**
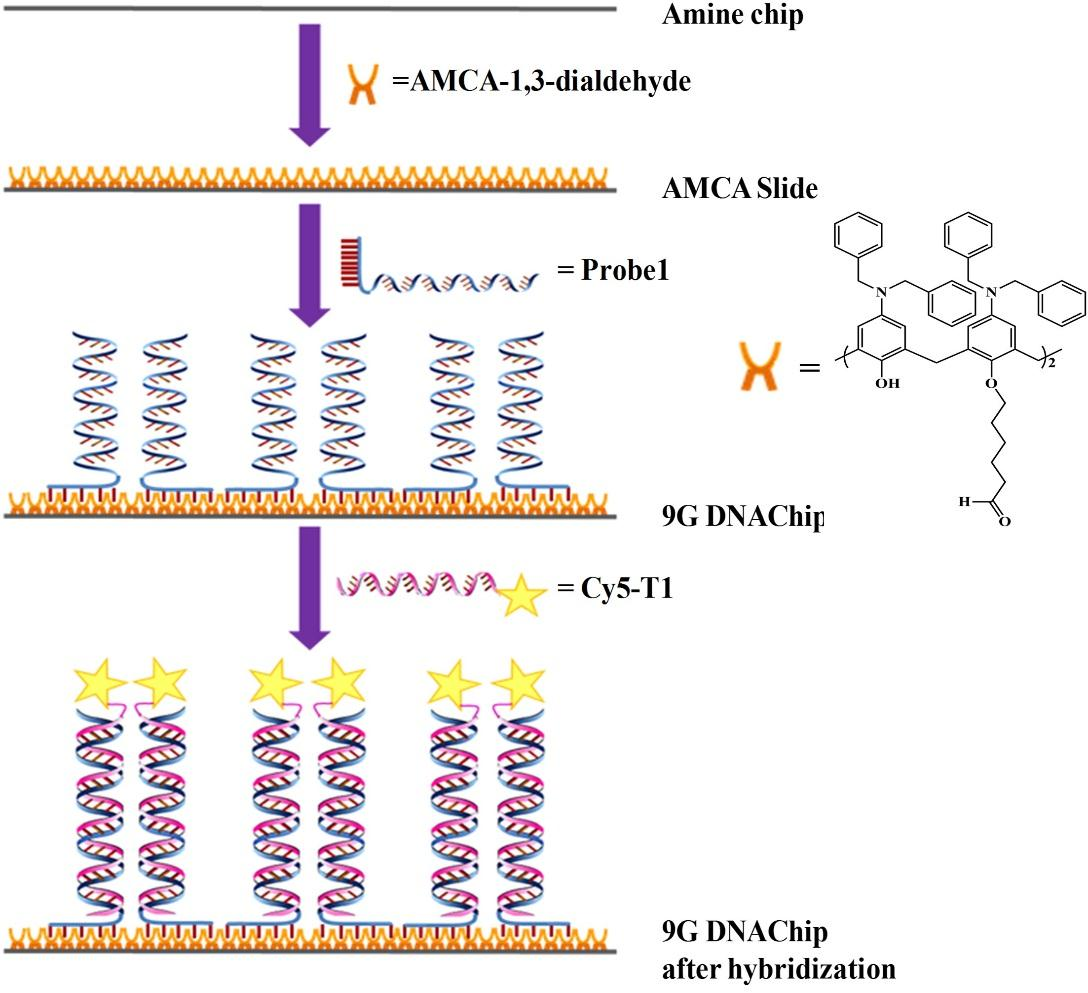
Preparation of 9G DNAChip and hybridization thereafter [81].

The preparation of the 9G DNAChip [81] and the hybridization thereafter is briefly explained in Figure 6. Aminocalix[4]arene (AMCA) slides were obtained by reacting an amine slide with AMCA-1,3-dialdehyde to generate a monolayer of AMCA on the surface. By spotting the solution of the oligonucleotides appended with nine consecutive guanines, the oligonucleotides can be immobilized on the AMCA slide to generate a 9G DNAChip. Cy5-labeled complementary oligonucleotides are hybridized and washed at 25 °C to evaluate the efficiency of 9G DNAChip.

Earlier, it was reported that the cavities of AMCA derivatives have a preference for structurally flat guests (like substituted aromatics, e.g., 4-methylpyridine, containing methyl groups (either a CH_3_ in the *para* position of an aromatic ring or the presence of a trimethylammonium group) [82–85]. Therefore, it was clear that the cavity of the aminocalix[4]arene shows strong molecular recognition with the structurally flat molecules such as the adenine, guanine, cytosine, and thymine in the DNA molecules.

To gain insight into the molecular recognition properties of the AMCA monolayer on the AMCA slide, further investigation was done by immobilization of the probes appended with nine adenine (9A), nine thymine (9T), nine guanine (9G), and nine cytosine (9C) subunits, respectively, on the AMCA slide. The 9T and 9C probes showed the lowest fluorescence intensity. The 9A probe also showed significant fluorescence intensity, but not as good as the 9G probe. Hence, oligonucleotides with nine consecutive guanine bases were used to generate 9G DNAChips. These findings pioneered the basis of the 9G technology.

The phenomenon of molecular recognition to immobilize oligonucleotides for the production of the DNA chips was first introduced based on the 9G technology. The 9G probes are immobilized by the multiple interactions of the nine consecutive guanines on the AMCA monolayer. The lateral spacing between the immobilized probes provides high accessibility leading to the more than 80% hybridization efficiency in 5 min at 25 °C. Moreover, the nine consecutive guanines can be easily added to the oligonucleotide probes during their synthesis. The excellent properties shown by the 9G DNAChip enables it to be a powerful and promising tool for nanotechnology, biotechnology and allied sciences.

## 3. Applications of Microarray Technology

Microarray technology has altered the scope of life science research and boosted the biomarker diagnostics industry [86,87]. Microarray technology has been extensively used for the detectionand discrimination of various pathogens and in the monitoring of antimicrobial resistant bacterial and viral strains [88]. The use of microarrays in the monitoring of host responses to infection and therapy, makes them the ultimate diagnostic platform for infectious diseases. The optimization of the diagnostic potential of microarrays and has led to the development of commercially available detection platforms. Thus, a new era in molecular diagnostics where the use of microarray technology in clinical microbiology has begun.

The regeneration of a surface-immobilized probes allows the reuse of DNA microarrays without the loss of hybridization activity. Thus, it can help in the reduction of the cost per test. However, for the reuse of the DNA microarrays three requirements should be taken into account with probe immobilization. Firstly, the immobilization chemistry needs to be stable, second the probes have to remain functional after attachment and lastly the DNAs have to be immobilized with an appropriate orientation and configuration [89].

The detection of protein biomarkers whose change in concentration with the progression of a disease is becoming increasingly important to monitor the right direction of treatment. However, use of the protein microarrays biomarker detection has been limited by issues such as the surface immobilization of proteins without loss in bioactivity [90,91]. To solve the several problems associated with protein microarrays, currently the DNA microarray methods are used for the detection of biomarkers. The following sections discuss some of the latest developments aimed at expanding the applicability of microarray biosensors for the detection of DNA and protein biomarkers for disease analysis.

### 3.1. Microarray Technology for Diagnosis of Viral/Bacterial Infections

In the last few years the development of new methods for the detection of infectious diseases such as human papillomavirus (HPV), human influenza virus (H1N1), pneumonia and tuberculosis has increased significantly [92]. Such infections necessitate a method for on-site diagnosis of infected individuals to not only administer effective treatment to affected patients, but also monitor on going prevention efforts, and improve opportunities for early therapy [93,94].

**Figure 7.**
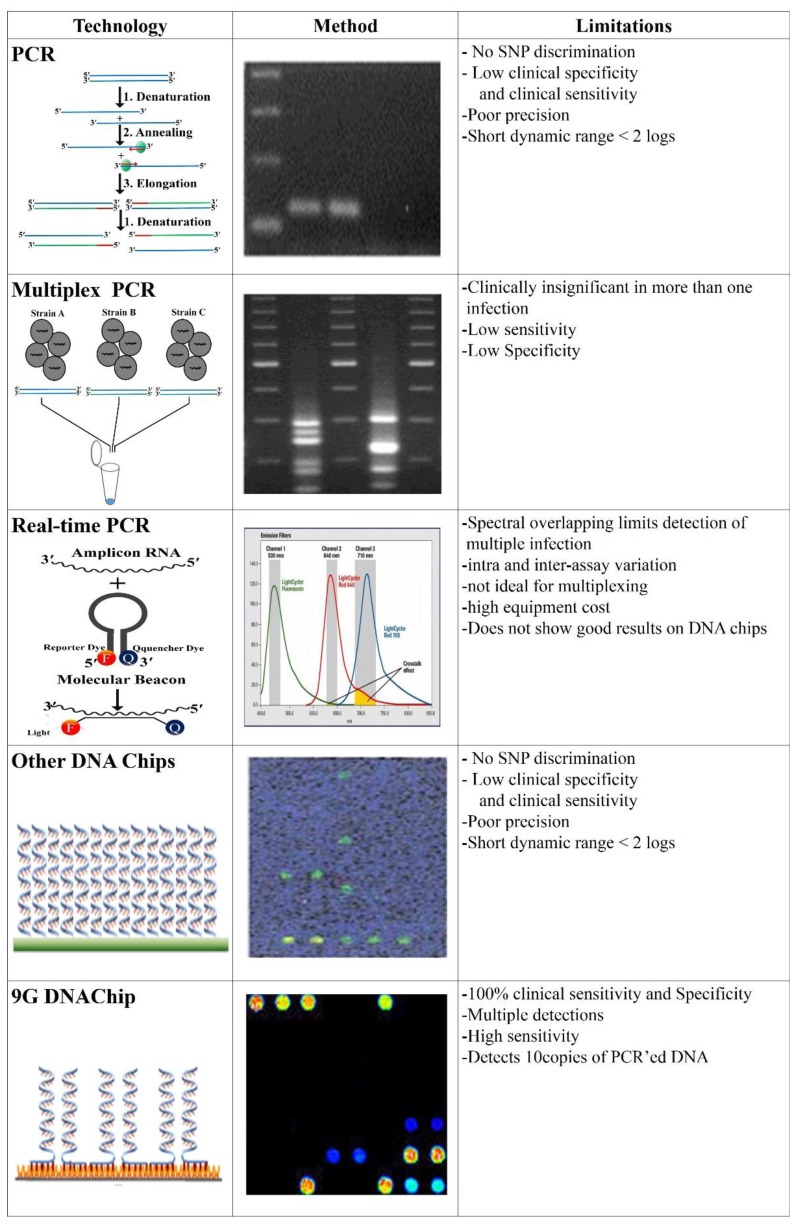
Advancements in the DNA detection technologies and their comparison.

As shown in Figure 7, there are several methods such as PCR [95], multiplex PCR [96], RT-PCR [97,98], which can screen the genotypes of the infectious agents. However, these PCR-based approaches suffer from drawbacks such as low clinical sensitivity and specificity, poor precision, inadequacy for multiplexing, and high equipment cost.

The limitation of the PCR technique is that it cannot discriminate the SNP efficiently and has a very low clinical sensitivity and specificity. Multiplex PCR, a much more powerful technique than PCR itself, also suffers from the problem of detection of multiple infections in a single sample and has a low sensitivity and specificity [99]. Although DNA chips [100,101] using pre-amplified PCR target DNAs for detection are more accurate as compared to the above methods since target DNAs bind with highly specific capture DNAs immobilized on the chip surface [102,103], most of the commercial microarrays suffer from the various problems such as low SBR, low SNP discrimination ratio, the necessity of high temperature hybridization (35∼45 °C), 100% target-specific hybridization, and very low clinical sensitivity and specificity. Therefore for accurate detection, a new on-site diagnosis method, capable of multiplex detection with high clinical sensitivity and specificity, is highly needed.

The 9G DNAChips obtained by the 9G DNAChip technology successfully solved these problems [104]. The 9G DNAChip show a high signal to background ratio (SBR), SNP discrimination ratio of 60:1, 100% target specific hybridization with more than 90% hybridization efficiency at 25 °C in less than 30 min, and 100% clinical sensitivity and specificity.

### 3.2. Microarray Technology for Biomarker Detection

**Figure 8.**
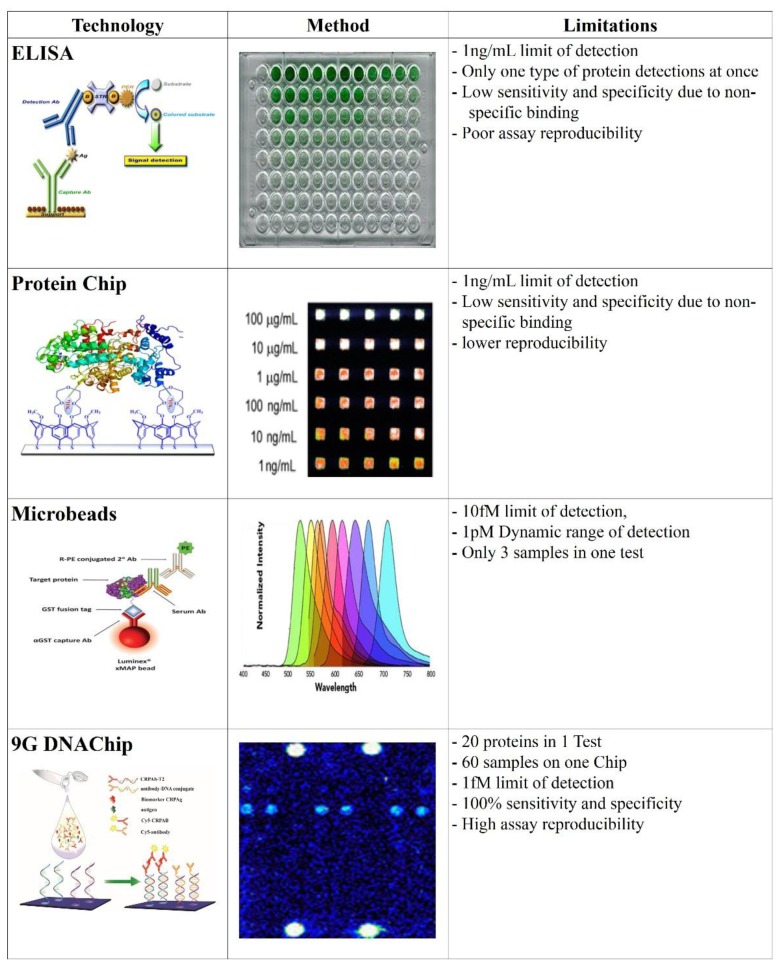
Advancements in the biomarker detection technologies and their comparison.

Enzyme-linked Immunosorbent Assays (ELISAs) are one of the earliest techniques employed for the detection of the biomarkers. Even though ELISA has several advantages for the detection of the biomarkers, it suffered from limitations as shown in Figure 8, which have triggered the development of protein chip technology.

**Figure 9.**
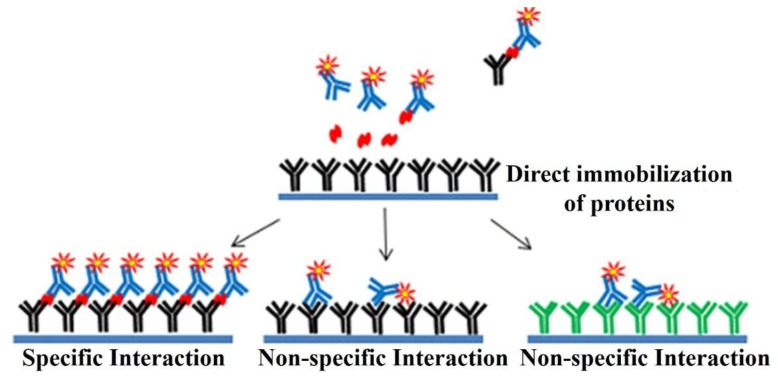
Detection of biomarkers with protein chips obtained by the direct immobilization methods.

Protein microarrays had been developed for the detection of the biomarkers [105]. The protein microarrays produced by the direct immobilization methods as shown in the Figure 9 are known to suffer from drawbacks like the instability of the immobilized proteins, and non-specific interactions, thus resulting in the low sensitivity [106–108].

DNA-Directed Immobilization (DDI) was employed to improve the stability of the proteins by immobilizing them on the surface shortly before the detection of antigens [109]. In the stepwise methods such as DDI, first the proteins are immobilized on the surface and in the second step they are allowed to couple with the target proteins. A common disadvantage of these methods is that the immobilized proteins have a chance to lose their activity over the period of time [110]. Thus, the sensitivity of these methods is limited to 100 pg/mL.

One of the drawbacks of the biomarker detection methods is that, their limit of detection is in the nanomolar range [111]. The other drawback of the conventional biomarker detection methods is that these methods can detect only one biomarker at a time, which limits the use of this technology [112]. The earlier reports on biomarker detection demonstrated that the conventional immobilization methods can also result in the loss of the activity of the analyte proteins [113]. However, the use of color coded beads in the xMAP^®^ technology of Luminex Corp. (Austin, TX, United States), allows multiplex analysis of samples. The beads used are color coded by different ratios of two fluorescent dyes [114,115].

According to the recently reported DNA-Guided Detection (DAGON) method [116] based on 9G technology, antigens with concentrations in the 1 pg/mL to 10 pg/mL range can be easily differentiated. The major difference between the DAGON and other methods is that the antigen–antibody biomolecular complexes are allowed to form in the solution. The biomolecular complex of the Cy5-labeled secondary antibody, the antibody–DNA conjugate and the target antigen formed in the solution is site-specifically guided to the predestined area on the chip surface to hybridize with the oligonucleotide probes at room temperature. Therefore, the DAGON method can detect multiple antigens in the mixture of the proteins with the concentrations of 1 pg/mL and 0.1 pg/mL without any amplification technique. The DAGON method shows 1000-fold improvement in the sensitivity as compared to the reported methods [117].

## 4. Future Directions

The 9G technology uses nine consecutive guanines in the immobilized probe which provides nanometer scale lateral spacing in the immobilized probes. However, by altering the number of guanine subunits in the immobilized probes, the lateral spacing between the immobilized probes can be controlled. Nanometer-sized patterns of biomolecules can be produced by using a known number of guanine residues in the immobilized probes. Thus, by hybridization of the immobilized probes with the conjugated target probes (protein-DNA conjugate, metal-DNA conjugate, cell-DNA conjugate, *etc.*) nanopatterns of the various molecules can been created on a surface.

Considering the wide scope of applications of the nanoscale positioning of different molecules on surfaces, we have a developed the generalized positioning system based on the 9G technology. Initial findings of the 9G technology for the positioning of molecules on the surface indicate that this technology will replace the traditional mixed self-assembled monolayer technique to fabricate the controlled molecular surfaces for various applications.

## 5. Conclusions

The clinical applications of the biosensors, biomolecular electronics, and nanodevices are greatly depend on the highly programmable positioning of molecules (biomolecules, nanoparticles, nanobeads, nanocomposites materials) on the surface. Unfortunately, conventional techniques such as self-assembled monolayers fail to position the molecules on a nanometer scale, which is a key to produce highly organized monolayers on the surfaces. The conventional techniques used for the immobilization of the probe DNA's and the proteins to produce molecular and biomarker diagnostic platforms suffer from the serious drawbacks. The drawbacks, such as crowding effect in immobilized probe DNA's, distortion of the three dimentional structure of the immobilized proteins limits the clinical applicability of micorarrays and the protein chips. Recently reported 9G DNAChips based on the 9G technology allow 80% hybridization in 5 min at 25 °C with 100% specificity. Therefore, the advantages and the drawbacks of conventional methods are compared with those of the 9G technology. It was noticed that the linear spacing between the immobilized probe DNAs plays a crucial role for the sensitivity and specificity of the microarray. We believe that the recent development in the chemical synthesis and modification of DNA will further allow to use the DNA as molecular building blocks for the highly functional microarrays.

## Figures and Tables

**Table 1.** Immobilization method of DNA probes on functionalized surfaces.

**Immobilization Method**	**Interaction or Reaction**	**Advantages**	**Drawbacks**	**Reference**
**Physical Adsorption**	Charge-charge interaction or Hydrophobic interaction	Simple	Desorption by change of ionic strength or pH	[32]
		Fast	Random orientation	
		Direct method (no linker molecules)	Desorption by detergent	
		Suitable to DNA, RNA, and PNA	Problem of crowding effect and poor reproducibility	
**Covalent bonding**	Chemical bonding	Good stability	Use of linker molecules	[33–36]
		High binding strength	Slow, Irreversible	
		Use during long term	Problem of crowding effect	
			Island formation	
**Streptavidin-Biotin interactions**	Specific Streptavindin-Biotin interaction	Improved orientation	Expensive, Slow	[37,38]
		High specificity and functionality	Problem of crowding effect	
		Well-controlled	Use of biocompatible linker	
		Reversible	Poor reproducibility	

**Table 2.** Immobilization method of functional DNA on functionalized DNA Chip surfaces.

**Surface Property**	**Group Structure**	**DNA Probe Modified**	**Immobilization Method**	**References**
Amine	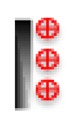	None	Physical absorption	[43]
Nitrocellulose	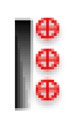	None	Physical absorption	[44]
Poly(l-lysine)	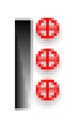	None	Physical absorption	[45,46]
PAAH	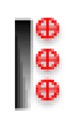	None	Physical absorption	[35]
Diazonium ion	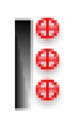	Non	Physical absorption	[47]
Gold (Au)	Au surface	Thiols (-SH)	Chemisorption	[48,49]
Carboxyl (with EDC)	-COOH group (with EDC)	Amines (-NH_2_)	Covalent	[50–52]
Aldehyde	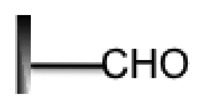	Amines (-NH_2_)	Covalent	[53–55]
Epoxy	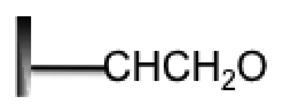	Amines (-NH_2_)	Covalent	[56]
Isothiocyanate	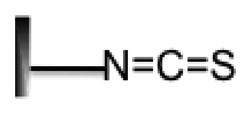	Amines (-NH_2_)	Covalent	[57,58]
Maleimide	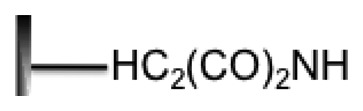	Thiols (-SH)	Covalent	[59]
Mercaptosilane	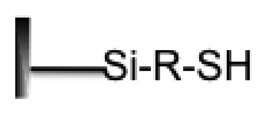	Thiols (-SH)	Covalent	[60]
Streptavidin	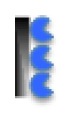	DNA-Biotin	Non-Covalent	[61,62]
Avidin	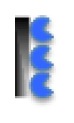	DNA-Biotin	Non-Covalent	[63]

**Table 3.** The advantages and drawbacks of functionalized DNA chip surfaces.

**Surface Function**	**Interaction or Reaction**	**Advantages**	**Drawbacks**
Carboxyl (EDC coupling)	Chemical bonding with amine-DNA	-Simple method of immobilization	-Efficiency of immobilization depends on pH, concentration, ionic strength, and reaction time.
		-High surface coverage of DNA's	
		-Easy coupling reaction	
Aldehyde	Chemical bonding with amine-DNA	-Good stability	-Long hybridization time
		-High binding strength	-Limits the absolute signal intensity
		-Stable enough for long term use	-High hybridization temperature
		-Less random immobilization	
Epoxy	Chemical bonding with hydroxyl, amine and sulfhydryl groups	-Easy protocol for immobilization	-Reactions between DNA and
		-Good stability	epoxy supports are extremely slow.
		-High binding strength	- Low Immobilization density
		-Stable enough for long term use	
Isothiocyanate	Chemical bonding amine-DNA	-Well-ordered surface	-High non-specific hybridizations
		-Re-usability	-Long hybridization time
		-High density DNA/area	
		-Stable enough for long term use	
Maleimide	Chemical bonding with sulfhydryl group of DNA	-Faster immobilization reaction	-Degradation in aqueous solutions
		-Good stability,	-High non-specific interaction
		-Re-usability	
		-High binding strength	
Mercaptosilane	Chemical bonding DNA-SH	-Good stability	-High non-specific interaction
		-Re-usability	-High hybridization temperature
		-High binding strength	
		-Stable enough for long term use	
